# The incidence of movement disorder increases with age and contrasts with subtle and limited neuroimaging abnormalities in argininosuccinic aciduria

**DOI:** 10.1002/jimd.12691

**Published:** 2023-12-04

**Authors:** Sonam Gurung, Saketh Karamched, Dany Perocheau, Kiran K. Seunarine, Tom Baldwin, Haya Alrashidi, Loukia Touramanidou, Claire Duff, Nour Elkhateeb, Karolina M. Stepien, Reena Sharma, Andrew Morris, Thomas Hartley, Laura Crowther, Stephanie Grunewald, Maureen Cleary, Helen Mundy, Anupam Chakrapani, Spyros Batzios, James Davison, Emma Footitt, Karin Tuschl, Robin Lachmann, Elaine Murphy, Saikat Santra, Mari‐Liis Uudelepp, Mildrid Yeo, Patrick F. Finn, Alex Cavedon, Summar Siddiqui, Lisa Rice, Paolo G. V. Martini, Andrea Frassetto, Simon Heales, Philippa B. Mills, Paul Gissen, Jonathan D. Clayden, Christopher A. Clark, Simon Eaton, Tammy L. Kalber, Julien Baruteau

**Affiliations:** ^1^ Great Ormond Street Institute of Child Health University College London London UK; ^2^ Centre for Advanced Biomedical Imaging University College London London UK; ^3^ Great Ormond Street Hospital for Children NHS Trust London UK; ^4^ Department of Clinical Genetics Cambridge University Hospitals Cambridge UK; ^5^ Mark Holland Metabolic Unit, Adult Inherited Metabolic Diseases Department Salford Royal NHS Foundation Trust Salford UK; ^6^ Willink Unit Manchester Centre for Genomic Medicine Manchester UK; ^7^ Evelina London Children's Hospital, St Thomas's Hospital London UK; ^8^ Charles Dent Metabolic Unit National Hospital for Neurology and Neurosurgery London UK; ^9^ Clinical IMD Birmingham Children's Hospital Birmingham UK; ^10^ Moderna, Inc. Cambridge Massachusetts USA; ^11^ National Institute of Health Research Great Ormond Street Biomedical Research Centre London UK

**Keywords:** ^123^I‐ioflupane, ammonia, argininosuccinate lyase, argininosuccinic aciduria, dopamine, movement disorder, nitric oxide, positron emission tomography, urea cycle

## Abstract

Argininosuccinate lyase (ASL) is integral to the urea cycle detoxifying neurotoxic ammonia and the nitric oxide (NO) biosynthesis cycle. Inherited ASL deficiency causes argininosuccinic aciduria (ASA), a rare disease with hyperammonemia and NO deficiency. Patients present with developmental delay, epilepsy and movement disorder, associated with NO‐mediated downregulation of central catecholamine biosynthesis. A neurodegenerative phenotype has been proposed in ASA. To better characterise this neurodegenerative phenotype in ASA, we conducted a retrospective study in six paediatric and adult metabolic centres in the UK in 2022. We identified 60 patients and specifically looked for neurodegeneration‐related symptoms: movement disorder such as ataxia, tremor and dystonia, hypotonia/fatigue and abnormal behaviour. We analysed neuroimaging with diffusion tensor imaging (DTI) magnetic resonance imaging (MRI) in an individual with ASA with movement disorders. We assessed conventional and DTI MRI alongside single photon emission computer tomography (SPECT) with dopamine analogue radionuclide ^123^I‐ioflupane, in *Asl*‐deficient mice treated by *hASL* mRNA with normalised ureagenesis. Movement disorders in ASA appear in the second and third decades of life, becoming more prevalent with ageing and independent from the age of onset of hyperammonemia. Neuroimaging can show abnormal DTI features affecting both grey and white matter, preferentially basal ganglia. ASA mouse model with normalised ureagenesis did not recapitulate these DTI findings and showed normal ^123^I‐ioflupane SPECT and cerebral dopamine metabolomics. Altogether these findings support the pathophysiology of a late‐onset movement disorder with cell‐autonomous functional central catecholamine dysregulation but without or limited neurodegeneration of dopaminergic neurons, making these symptoms amenable to targeted therapy.

## 1 INTRODUCTION

Argininosuccinic aciduria (ASA) (OMIM#207900) is a rare autosomal recessive metabolic disease, with a prevalence of 1 in 110 000 live births and is the second most common urea cycle disorder.^1^ Patients present with acute hyperammonemia either in the neonatal period (early‐onset phenotype), or later in life (late‐onset presentation).^2^ Patients can develop a systemic phenotype with chronic neurological (50%–92%), hepatic (37%–52%) and gastrointestinal (33%) problems, hypokalaemia (46%) and arterial hypertension in isolated cases.^3^, ^4^, ^5^, ^6^, ^7^, ^8^, ^9^ The neurological phenotype is variable with intellectual difficulties, attention deficit, epilepsy, behavioural changes and a movement disorder.^3^, ^10^, ^11^ Whilst hyperammonemia can explain some of the symptoms and the neurological sequelae observed in ASA chronic encephalopathy and these symptoms often occur despite satisfactory control of ammonia levels.^3^, ^12^ The best‐accepted therapy for ASA relies on ammonia control through use of a protein‐restricted diet, ammonia scavengers and arginine supplementation^2^, ^13^ with an increasing number of patients treated by liver transplantation.^14^ It has been suggested recently that liver transplantation could have a sustained neurological benefit even in individuals with ASA with well‐controlled ammonia levels.^15^

ASA is caused by the deficiency of the cytosolic enzyme argininosuccinate lyase (ASL), the only mammalian enzyme enabling endogenous arginine synthesis.^16^ ASL breaks down argininosuccinic acid into arginine and fumarate. This biochemical reaction is integral to the nitric oxide (NO) cycle, facilitating NO synthesis from arginine, and the urea cycle, a liver‐based pathway enabling excess nitrogen to be converted to ammonia prior to conversion to urea for excretion.^17^ The pathophysiology of ASA chronic encephalopathy is likely multifactorial with detrimental roles of hyperammonemia, argininosuccinate toxicity,^18^ deficiency of arginine and downstream metabolites such as creatine,^1^ neuronal nitro‐oxidative stress^19^ and NO‐mediated downregulation of central catecholamine biosynthesis suggesting a neurodegenerative phenotype.^20^, ^21^

Here, we present a national multicentre retrospective study assessing the movement disorder phenotype in ASA, a well‐recognised feature of ASA chronic encephalopathy. We provide a clinical description of neurodegenerative‐related symptoms, movement disorder, hypotonia/fatigue and abnormal behaviour for a large cohort of patients, present patient neuroimaging data and highlight the difficulties in modelling the movement disorder phenotype in ASA mouse models. We show that the incidence of movement disorder in ASA increases with ageing, starting in adolescence and early adulthood. Abnormal diffusion tensor imaging (DTI) features can affect both the grey and white matter, preferentially close to basal ganglia. In order to assess the role of cerebral ASL deficiency (ASLD) without the confounding bias of hyperammonemia, liver‐targeting mRNA therapy‐treated ASLD mice with normalised ureagenesis were assessed. They did not recapitulate the DTI patient findings and showed normal single photon emission computer tomography (SPECT) with ^123^I‐ioflupane, a dopamine analogue radionuclide. These findings support the pathophysiology of a movement disorder in ASA with functional central catecholamine dysregulation but without loss of dopaminergic neurons, making movement‐disorder symptoms amenable to targeted therapy.

## 2 MATERIALS AND METHODS

### 2.1 Patients

We conducted a retrospective study in six paediatric and adult tertiary metabolic centres in the UK. Electronic records from July 2015 to June 2022 of epidemiological and clinical data of individuals with ASA with neurological disease were reviewed retrospectively by a physician. A patient was considered as having the symptom even if this feature was identified but not present at subsequent or the latest visit.

Movement disorder‐related symptoms were considered when reported by a patient during medical history investigation or when observed during medical examination. The following symptoms were considered: (i) involuntary abnormal movements such as tremor or ataxia, excluding seizures, (ii) abnormal muscle tone, unusual and unexplained acute episodes of fatigue and chronic lethargy, (iii) early signs observed in neurodegenerative diseases such as sleep disturbances and abnormal behaviour.

Abnormal behaviour was considered as a generic term when a patient presented with aggressive behaviour or sleep disturbances. Aggressive behaviour was either self‐harming or hetero aggressivity requiring intervention from a professional (e.g., psychological or emotional support from a psychologist, medication prescribed by a physician) or adaptation of the environment (e.g., self‐protective equipment, specific management protocol at school or work).

Myopathic features were defined as tiredness, fatigue, hypotonia and myopathy‐like symptoms. Acute tiredness and chronic fatigue were used for profound lethargy and unexplained episodes of general weakness persisting several days before spontaneous recovery. Acute tiredness and chronic fatigue were used for symptoms lasting for less and more than 2 weeks, respectively.

Intellectual disability was determined by clinical judgement of the metabolic specialist or paediatric neurologist or by the need for additional support at school or in the workplace.

Early‐onset ASA was defined as hyperammonemia occurring in the patient or familial index case on or before 28 days of age, and late‐onset ASA as hyperammonemia occurring after 28 days of age.

The human *ASL* Refseq transcript ID NM_000048.4 was used as the reference sequence when genotyping the patients.

### 2.2 Patient's neuroimaging analysis

A brain magnetic resonance imaging (MRI) was performed in one individual with ASA. The standard operating procedures of Great Ormond Street Hospital for Children were followed for conventional T_1_‐weighted and diffusion tensor imaging (DTI) sequences. Images were obtained using a Siemens Prisma 3T MRI scanner (Erlangen, Germany) and a 20‐channel head coil. T_1_‐weighted images were acquired using an MPRAGE sequence with a repetition time (TR) of 2300 ms, time to echo (TE) of 2.74 ms, field of view (FOV) of 256 × 256 mm, 1 mm slices and a 256 × 256 matrix. The DTI sequence consisted of a multiband spin‐echo echo planar imaging sequence with 60 directions at *b* = 1000 s/mm^2^ and 60 directions at *b* = 2200 s/mm^2^, with 13 *b* = 0 images interleaved throughout the sequence and an additional *b* = 0 image with negated phase‐encoding for distortion correction, TR of 3050 ms, TE of 60 ms, FOV of 220 × 220 mm, a 110 × 110 matrix, 2 mm slices with a 0.2 mm slice gap and multiband factor 2. Basal ganglia volumes were derived from FreeSurfer^22^ (caudate, putamen and pallidum bilaterally) using an analysis of covariance with age, gender and total intracranial volume as covariates. Statistical analysis of fractional anisotropy (FA) and mean diffusivity (MD), averaged over each subcortical regions of interest (ROI) was performed using an analysis of covariance with age and gender as covariates. A multiple comparisons correction was applied using false discovery rate (FDR).

### 2.3 mRNA formulation

*hASL* and Luciferase (*Luc*) encoding mRNA encapsulated in lipid nanoparticles (LNPs) were provided by Moderna Therapeutics using their proprietary technology. Codon optimised mRNA encoding *hASL* was synthesised in vitro by T7 RNA polymerase‐mediated transcription. The mRNA initiated with a cap, followed by a 5′‐untranslated region (UTR), an open reading frame encoding *hASL*, a 3′‐UTR and a polyadenylated tail. Uridine was globally replaced with N1‐methylpseudouridine, as described previously.^23^ LNP formulations were generated for in vivo intravenous (IV) delivery.^24^ In brief, mRNA was mixed with lipids in a molar ratio of 3:1 (mRNA:lipid). mRNA‐loaded nanoparticles were subsequently diluted into final storage buffer and had particle sizes of 80–100 nm, >80% encapsulation of the mRNA by RiboGreen assay and <10 EU/mL endotoxin levels.

### 2.4 Animals

Animal procedures were approved by institutional ethical review and performed per UK home office licences PP9223137 and PEFC6ABF1. *Asl* ^*Neo/Neo*^ mice (B6.129S7‐*Asl* ^*tm1Brle*^/J) were purchased from Jackson Laboratory (Bar Harbour, ME) and maintained on standard rodent chow (Harlan 2018, Teklab Diets, Madison, WI) with free access to water in a 12‐h light/12 h dark environment. Wild‐type (WT) littermates were used as controls and housed in the same cages. Genotyping was performed using DNA extracted from tail clips as described previously.^19^

### 2.5 Animal experimental design

*Asl* ^*Neo/Neo*^ animals received systemic administration of *hASL* mRNA from birth then weekly until the age of 8 weeks at a dose of 1 or 2 mg/kg for IV and intraperitoneal (IP) injections, respectively. Untreated WT littermates were used as controls. All *Asl* ^*Neo/Neo*^ animals survived with normal growth and fur as described previously.^25^

### 2.6 Magnetic resonance imaging in mice

Images were acquired on a 9.4 Tesla Bruker imaging system (BioSpec 94/20 USR) with a horizontal bore and 440 mT/m gradient set with an outer/inner diameter of 205 mm/116 mm, respectively (BioSpec B‐GA 12S2), 86 mm volume coil, and a four‐channel array receiver‐surface coil (RAPI Biomedical GmbH). The brain ROI were first localised using a structural T2‐TurboRARE sequence (fast‐spin echo, Paravision 7.0). The olfactory bulbs were used as an anatomical landmark to maintain consistency in slice positioning between subjects and the slices covered the cortex and all subcortical structures up to the cerebellum. Imaging parameters for the T_2_‐weighted imaging sequence were as follows: TR = 4000 ms, TE = 45 ms, FOV = 21 × 16 mm^2^, data matrix 256 × 196, 14 × 600 μm coronal slices.

Diffusion weighted imaging (DWI) was performed using a 4‐shot spin echo‐planar imaging sequence. Imaging parameters were: TR = 2500 ms; FOV = 20 × 16 mm^2^; FOV = 20 × 16 mm^2^, data matrix 110 × 85; 14 × 600 μm coronal slices. To implement the multiple echo‐time neurite orientation dispersion and density imaging model (MTE‐NODDI), the DWI images were acquired at three different echo times of 30, 45, and 60 ms. At each echo time, the MRI protocol consisted of two shells, detailed as follows:

1. Shell one: 30 directions, five *b* = 0 s/mm^2^ images and diffusion weighting of *b* = 2000 s/mm^2^
2. Shell two: 15 directions, five *b* = 0 s/mm^2^ images and diffusion weighting of *b* = 700 s/mm^2^

with gradient duration and separation *δ*/*Δ* = 4.5/11 ms for all *b*‐values and TEs. The total acquisition time was approximately 120 min.

### 2.7 Image processing and quantification of diffusion data

The effects of noise and imaging artefacts on the acquired diffusion data were reduced by applying a denoising method based on random matrix theory (MRtrix3), correction of B0 inhomogeneity and motion with TOPUP tool in FMRIB Software Library (FSL, University of Oxford, UK). DWI images were then co‐registered to a reference *b* = 0 image. Brain masks were created manually using ITK‐SNAP software (www.itksnap.org).^26^ Standard DTI measures of FA and MD were generated from diffusion data obtained at TE = 30 ms using DTIfit in FSL, which fits a diffusion tensor model at each voxel of the data that has been pre‐processed. NODDI parameters were estimated for each TE session separately with the NODDI MATLAB toolbox, and the estimated parameters were aligned to the first TE session to extract TE‐independent MTE‐NODDI parameters.

For quantitative analysis, brain ROIs were manually defined using ITK‐SNAP. ROIs were drawn in WT and *hASL* LNP‐mRNA treated Asl^Neo/Neo^ mice in the cortex, striatum for grey matter; corpus callosum, fimbria, and internal capsule for white matter; and hippocampus as a function region. All ROIs were subsequently exported to fitted diffusion maps and mean values for neurite density index (NDI), extra‐neurite volume fraction (*f*_en_), orientation dispersion index (ODI), FA and MD were exported for quantitative analysis.

### 2.8 SPECT imaging in mice

^123^I‐ioflupane was obtained from Curium Pharma UK Ltd with a patient dose of 185 MBq. Mice were injected with approximately 15–20 MBq of ^123^I‐ioflupane via tail vein injection. Mice were then anaesthetised using isoflurane anaesthesia (1.5%–2% isoflurane in oxygen 1 L/min) and mouse head SPECT/CT scans were acquired 2 h after injection using a NanoScan SPECT/CT system (Mediso, Hungary). CT images were acquired using a 55 KV peak (kVp) x‐ray source with 300 ms exposure time in helical mode, resulting in a scan time of approximately 3–4 min. SPECT images were obtained with a four‐head scanner with nine 1.4 mm pinhole apertures in helical scan mode using a time per view of 60 s resulting in a scan time of 40 min. Respiration was monitored throughout the scan and the body temperature was maintained by a heated bed. CT images were reconstructed in voxel size 124 × 124 × 124 μm using Mediso Nucline (Mediso, Hungary) software, whereas SPECT images were reconstructed in a 256 × 256 matrix using HiSPECT (ScivisGmbH, Bioscan/Mediso). Images were analysed using VivoQuant software (InViCro). 3D ROIs were drawn manually and included the whole brain, a spherical region including the locus coeruleus, and basal ganglia. The percentage of injected dose/organ (%ID/organ) was calculated using decay‐corrected ROI values.

### 2.9 Dopamine metabolites

Dopamine, 3‐O‐methylDOPA, homovanillic acid, 3,4‐dihydroxyphenylacetic acid and 5‐hydroxyindolacetic acid (5‐HIAA) were quantified using reverse‐phase high performance liquid chromatography as described previously.^27^

### 2.10 Statistical analysis

Statistical analysis was performed using Prism 9.0 software (San Diego, CA, USA). Differences between groups were assessed using a two‐tailed unpaired *t* test with adjustment for multiple comparisons using FDR. *p* values ≤0.05 were considered statistically significant. Correlation between continuous variables was assessed by Spearman's rank correlation test.

## 3 RESULTS

### 3.1 Demographic and clinical characteristics

Sixty patients (32 males, 28 females) were included with a median age of 12.7 years (range: 6 months to 53 years). Thirty‐four patients (57%) had early‐onset ASA. ASA was diagnosed biochemically and was confirmed genetically in 25 patients (Table S1). Three patients who died during the first month of life were excluded from the analysis.

### 3.2 Clinical characteristics of movement disorder‐related symptoms

This study included 17 (28%) individuals with ASA with neurodegenerative‐related symptoms, movement disorder, hypotonia/fatigue and abnormal behaviour. The median age was 19 years (range: 4–53 years) and the ratio of male:female was 10:7 (Table 1).

**TABLE 1:** Features of individuals with argininosuccinic aciduria with movement disorder‐related symptoms.

Patient number	Form	Sex	Survival (age at death)	Age at last follow‐up (years)	Long‐term neurological phenotype								Hyperammonaemic episodes	Genotype
					Abnormal movements		Hypotonia		Behaviour		Intellectual disability	Epilepsy	Yes/no	
1	Early	F	Alive	20	Y (tremor, ataxia, dysdiadochokinesia)	11 years	Y (fatigue)	10 years	N	–	Y	N	Y	NA
2	Early	M	Alive	4	N	–	Y (acute tiredness)	1 years	N	–	Y	N	Y	c.1045‐1057del; c.1045‐1057del
4	Early	M	Alive	12	Y (tremor)	9 years	Y (myopathic face, fatigue)	9y	Y (aggressivity, sleep disorder)	17 years	Y	Y	Y	c.719‐2A>G; c.857A>G
5	Early	M	Alive	11	N	–	Y (acute tiredness)	11 years	N	–	Y	Y	Y	c.437G>A; c.437G>A
6	Early	F	Died (12 years)	13	N	–	Y (fatigue)	13 years	N	–	Y	N	Y	NA
8	Early	M	Alive	18	N	–	Y (fatigue)	18 years	N	–	Y	Y	Y	c.1153C>T; c.1153C>T
9	Early	M	Alive	25	Y (dystonia)	25 years	N	–	N	–	Y	Y	Y	c.1153C>T; c.1153C>T
14	Early	F	Alive	10	Y (ataxia)	10 years	N	–	N	–	Y	N	Y	NA
24	Late	M	Died (53 years)	53	Y (nystagmus, ataxia)	NA	Y (swallowing difficulties)	NA	N	–	Y	Y	Y	NA
25	Late	M	Alive	24	Y (ataxia, tremor)	24 years	N	–	N	–	Y	Y	Y	c.437G>A; c.446+1G>A
27	Late	F	Alive	28	N	–	N	–	Y (aggressivity)	28 years	Y	N	Y	NA
28	Late	M	Alive	16	N	–	Y (acute tiredness, fatigue, ptosis, myopathic face)	12 years	N	–	Y	Y	Y	NA
31	Late	M	Alive	19	N		Y (fatigue)	15 years	Y (aggressivity)	16 years	Y	N	Y	NA
33	Late	F	Alive	26	Y (ataxia)	8 years	N	–	N	–	Y	Y	Y	c.348+1G>A; c.532G>A
35	Late	F	Alive	22	Y (ataxia)	NA	N	–	N	–	Y	N	N	NA
36	Late	M	Alive	20	Y (ataxia)	9y	N	–	N	–	Y	N	N	NA
47	Early	F	Alive	15	N	–	N	–	Y (aggressivity, sleep disorder)	10 years	N	N	N	NA

*Note*: For abnormal movement, hypotonia and behaviour, the age mentioned refers the age of onset of these symptoms.

Abbreviations: F, female; M, male; N, no; NA, not available; Y, yes.

**FIGURE 1:**
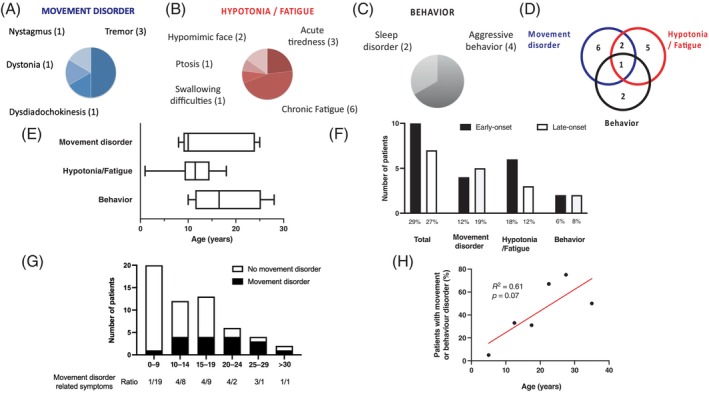
Movement, hypotonia/fatigue and behaviour disorders in individuals with argininosuccinic aciduria (ASA). (A) Individuals with ASA can present symptoms affecting (A) movement disorders, (B) hypotonia/fatigue and (C) behaviour. (D) These symptoms can be isolated or overlapping in one individual. (E) These symptoms are not present during the first decade and appear during the second and third decades. (F) The frequency of affected patients for these symptoms is similar between early‐ and late‐onset ASA patients. (G) The ratio of individuals with ASA presenting with movement disorder, hypotonia/fatigue and/or behaviour is presented according to age range. (H) Simple linear regression between the percentage of individuals with ASA presenting with movement disorder, hypotonia/fatigue and/or behaviour against the age range of patients.

A movement disorder phenotype and hypotonia/fatigue were the most frequent symptoms observed. Movement disorder was observed in nine (15%) patients with a median age at onset of 10 years (range: 8–25 years) with a ratio of male:female of 5:4 (Figure 1A). Tremor was observed in three patients and described as intention tremor, with high amplitude, rhythmic and oscillatory tremor during a purposeful movement. No resting tremor was observed. Patients with tremor reported a negative impact on their quality of life. No patient received tremor‐targeting therapy. One patient presented with dystonia at the age of 25 years. Hypotonia and fatigue‐related symptoms were observed in nine (15%) patients with a median age at onset of 11.5 years (range: 1–18 years) and a male:female ratio of 7:2 (Figure 1B). Behaviour changes were observed in four (7%) patients with a median age at onset of 16.5 years (range: 10–28 years) and a male:female ratio of 1:1 (Figure 1C). Some patients presented with a combination of these symptoms (Figure 1D). After a symptom free interval during childhood, the onset of hypotonia/fatigue‐related symptoms and behaviour changes was observed in the second decade, whereas movement disorder symptoms were recognised in early adulthood during the third decade (Figure 1E). These symptoms affected early‐ and late‐onset patients similarly (Figure 1F). The prevalence of these symptoms increased gradually with ageing with 100% of patients aged 25–29 years affected (Figure 1G). Patients aged >30 showed a lower prevalence with only 33% displaying these symptoms. A correlation between prevalence of these symptoms and age was observed (Spearman's correlation coefficient *r* ^2^ = 0.22, *p* = 0.34) (Figure 1H).

Individuals with ASA presenting with neurodegeneration‐related symptoms did not have a higher risk of learning disability (*p* = 1) and epilepsy (*p* = 0.79), compared to individuals with ASA without a movement disorder phenotype.

Only one patient with neurodegenerative‐related symptoms had not had previous episodes of hyperammonemia (Table 1).

### 3.3 Brain MRI in an individual with ASA showed normal conventional sequences but functional MRI revealed involvement of basal ganglia

We aimed to assess neuroimaging in one patient with ASA who had neurodegenerative‐related symptoms and which included a movement disorder phenotype. A neonatal‐onset male ASA patient was investigated at the age of 16 years using brain MRI with conventional and diffusion tensor imaging (DTI) sequences. Their diagnosis had been confirmed by identification of bi‐allelic pathogenic mutations in the *ASL* gene (c.719‐2A>G and c.857A>G [p.Q286R]) and their symptoms were managed with a protein‐restricted diet, sodium benzoate (200 mg/kg/day) and arginine (70 mg/kg/day) supplementation. He had previously had greater than five hyperammonemic decompensations during childhood and adolescence and had participated in a phase I/II clinical trial of cell therapy (NCT01765283). The latter was not shown to be efficacious, it did not improve ureagenesis and did not allow therapies based on standard of care to be relaxed. The patient had mild learning difficulties, with intention tremor, a hypomimic face and occasional episodes of unexplained and self‐resolving acute tiredness. A brain MRI with conventional T_1_‐weighted and diffusion tensor imaging (DTI) sequences was performed at the age of 16 years to explore his motor disorder.

**FIGURE 2:**
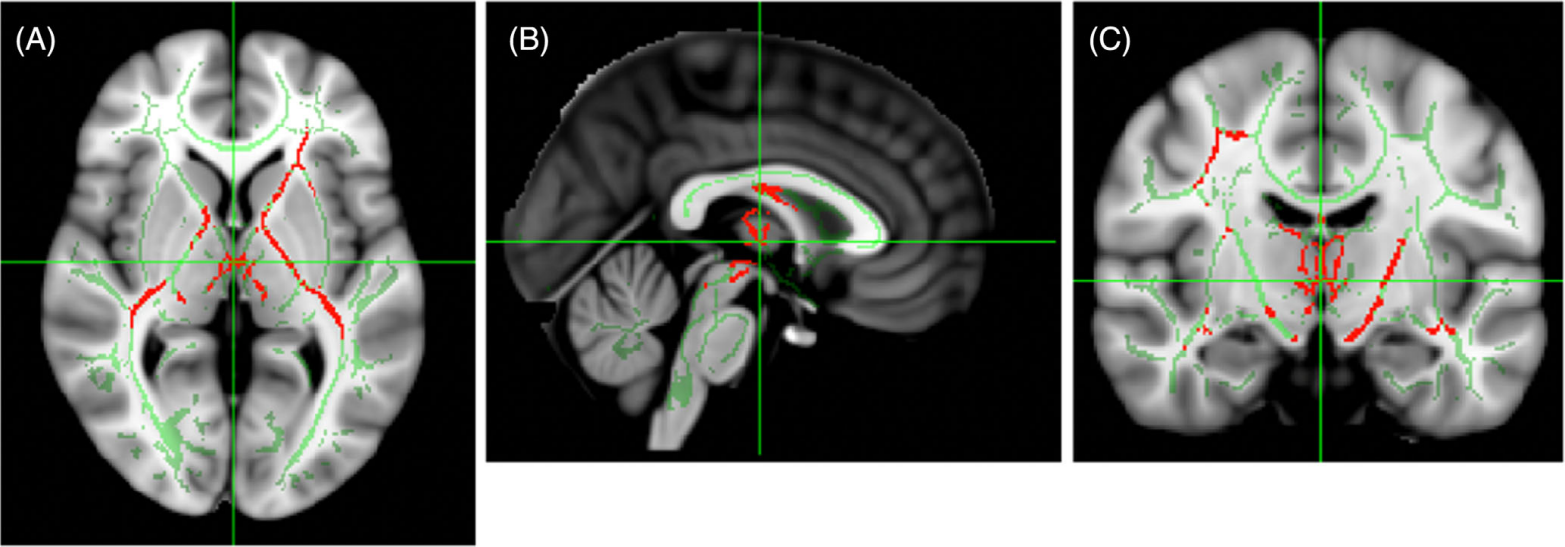
Functional magnetic resonance imaging from an individual with argininosuccinic aciduria (ASA) showed involvement of basal ganglia. (A–C) Decreased fraction of anisotropy in the internal capsule, fornix and white‐matter tracts in the vicinity of basal ganglia in an individual with ASA with (A) axial, (B) sagittal and (C) coronal sections.

A global and detailed analysis of the brain was performed using conventional and functional MRI, with a focus on basal ganglia areas involved in movement disorder phenotypes. This was compared to age and sex‐matched control cohort images (*n* = 21: 7 males and 14 females, age 16.7 ± 1.7 years). There was no difference in the volume of the caudate, putamen and pallidum of the individual with ASA and the control cohort. A comparison however, of the more focussed DTI MRI analysis of the subcortical ROI showed a significantly higher MD for both left and right pallidum in the individual with ASA, compared to controls (*p* = 0.0006 and *p* = 0.0022, respectively) as well as the left and right putamen (*p* = 0.0037 and *p* = 0.0041, respectively). No difference in FA was observed in the individual with ASA compared to controls in any of the ROI. A tract‐based spatial statistics analysis^28^ was performed to analyse the white matter microstructure. The patient has significantly lower FA than controls (*p* < 0.05) bilaterally in the internal capsule, external capsule and cerebral peduncle, as well as the left anterior corona radiata and fornix (Figure 2A–C). No significant difference in the MD for the white matter was detected.

### 3.4 Functional MRI in LNP‐mRNA‐treated ASA mice

**FIGURE 3:**
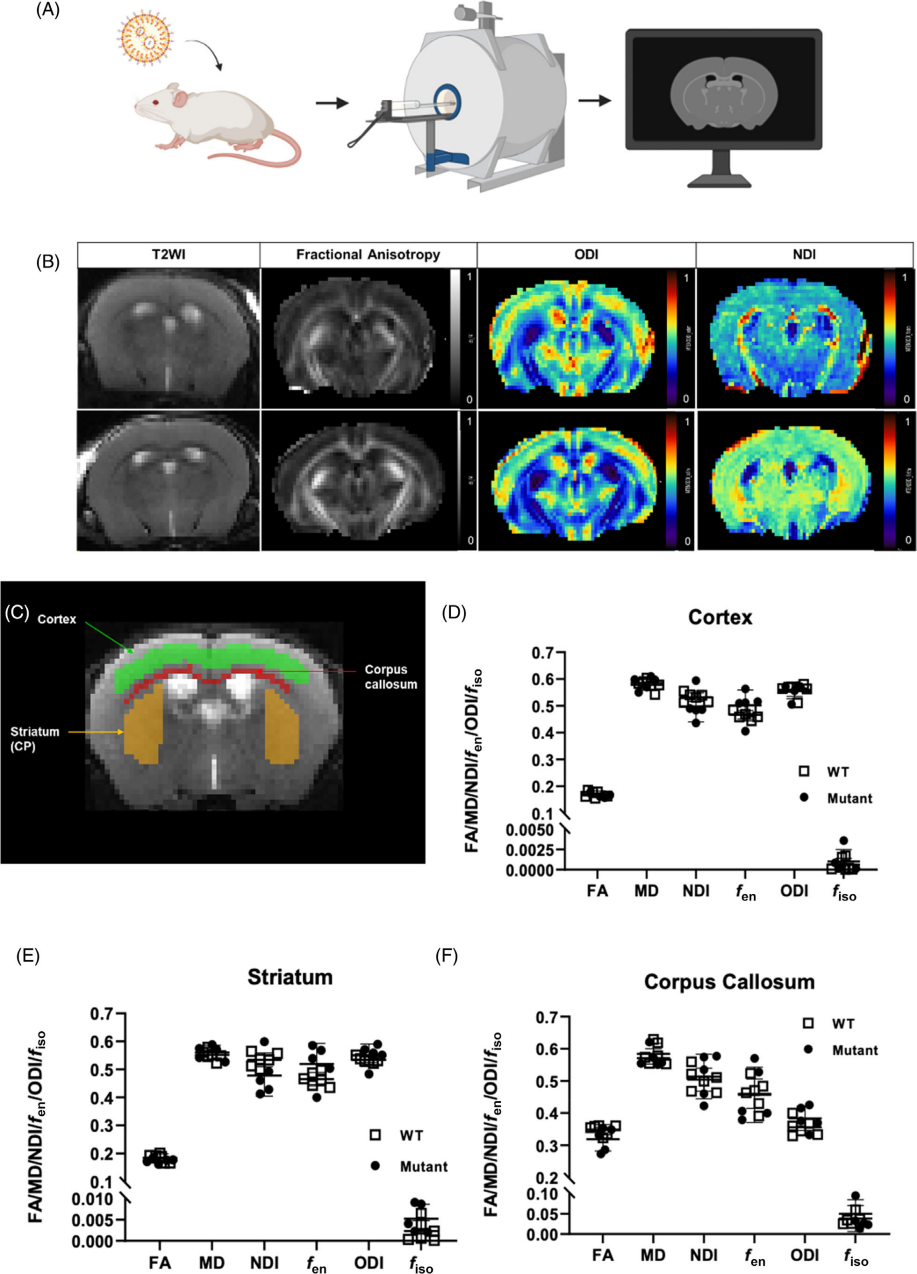
Functional magnetic resonance imaging (MRI) in lipid nanoparticles (LNP)‐mRNA treated argininosuccinic aciduria mice. (A) Schematic describing the experimental study in *Asl* ^*Neo/Neo*^ mice treated systemically by *hASL* LNP‐mRNA from birth until 7 weeks of age. The MRI was performed 3–4 days after the last injection of *hASL* LNP‐mRNA, before post‐processing and data analysis. (B) Representative MRI images of *hASL* LNP‐mRNA treated *Asl* ^*Neo/Neo*^ mice with conventional T2, fractional anisotropy (FA), orientation dispersion index (ODI) and neurite dispersion index (NDI) sequences. (C) Manual definition of cerebral regions of interest with ITK‐SNAP software. (G–I) Analysis of functional magnetic resonance imaging endpoints in various cerebral regions of interest (D) cortex, (E) striatum and (F) corpus callosum. Graph shows mean ± SD. (D–F): Unpaired two‐tailed Student's *t* test (false discovery rate corrected; *p* = 0.05, *n* = 5–6; ns, not significant. MD, mean diffusivity.

Movement disorder in ASA is, at least partially, not related to hyperammonemia as demonstrated in a knockout *Asl* model in dopaminergic neurons, *Asl* ^*flox/flox*^;*TH Cre* ^*+/−*^ mouse.^20^, ^21^ Various neurological symptoms in individuals with ASA appear unrelated to hyperammonemia such as developmental delay,^3^ epilepsy^12^ and tremor.^20^ Therefore, we aimed to assess the cell‐autonomous central component of the neurological disease in an ASLD mouse with normalised ureagenesis to exclude the potential bias of hyperammonemia. Modelling movement disorder‐related symptoms is essential to better understand the pathophysiology of the disease, identify the main cerebral structures affected and test adequate therapies. In order to test the hypothesis that the ureagenesis defect is not the main cause of the motor phenotype in ASLD, the lethal *Asl* ^*Neo/Neo*^ mouse model, which is recognised as a reliable model recapitulating the ASLD human phenotype, was maintained by systemic administration of the liver‐targeting *hASL* LNP‐mRNA formulation weekly from birth until adulthood. This normalised plasma ammonia (Figure S1A), ALT (Figure S1B) levels and liver ASL activity (Figure S1C). The ASL biodistribution in the liver of *hASL* mRNA treated *Asl* ^*Neo/Neo*^ mice is homogeneous across the whole hepatic lobule.^25^ At 8 weeks of age, these ASLD mice underwent brain MRI with both structural and DWI sequences before downstream modelling and quantification (Figure 3A). The images obtained were of sufficient quality to reliably delineate the different brain structures and assess multiple parameters including volume on conventional T_2_‐weighted images and standard DTI metrics such as FA, MD. We also used the NODDI model to extract ODI, *f*_en_, NDI and free‐water volume fraction (isoVF) which describe underlying cerebral microstructure more accurately than DTI (Figure 3B). Different ROI were drawn (Figures 3C and S2A) and analysed: frontal cortex (Figure 3D), striatum (Figure 3E), fimbria (Figure S2B) and hippocampus (Figure S2C) to represent grey matter; corpus callosum (Figure 3F), and internal capsule (Figure S2D) to assess white matter. In contrast to the patient's data (Figure 2A–C), no difference between WT and *hASL* LNP‐mRNA‐treated Asl^Neo/Neo^ mice was observed (Figures 3D–F and S2B–D).

### 3.5 Dopamine metabolism in LNP‐mRNA treated ASA mice

**FIGURE 4:**
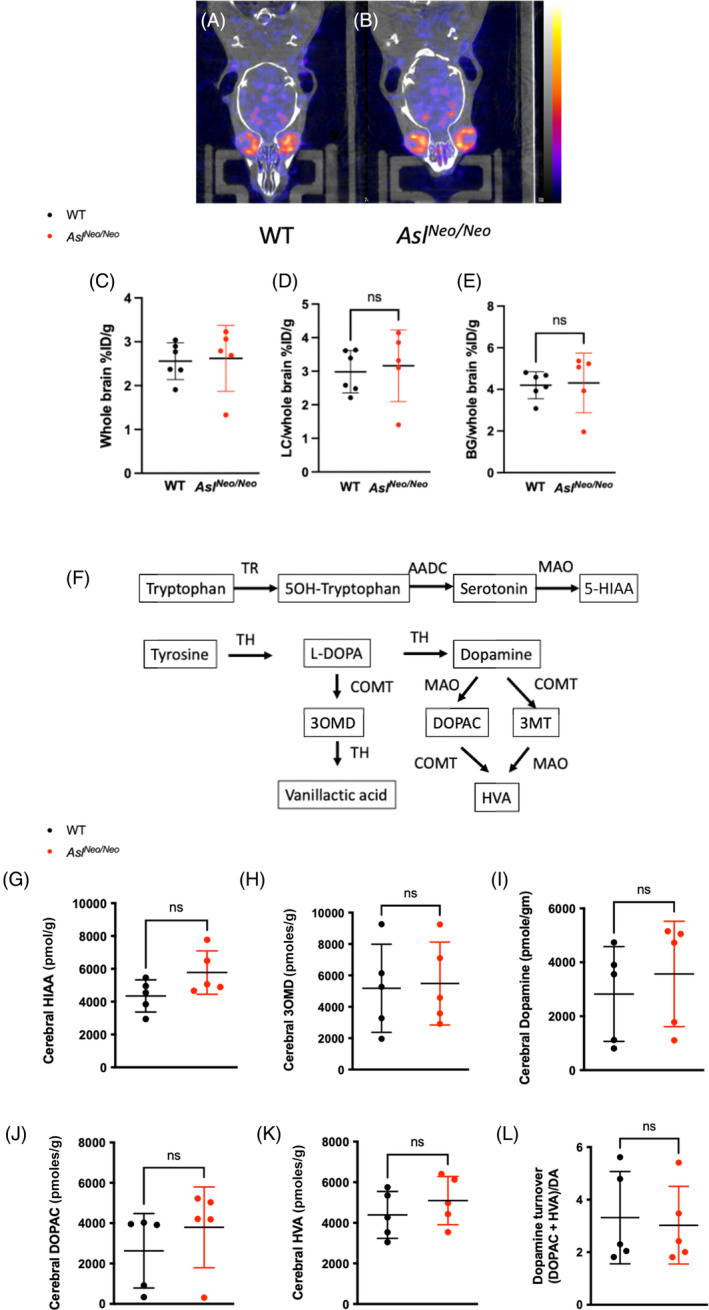
Dopamine metabolism in lipid nanoparticles (LNP)‐mRNA treated AS mice. (A, B) Representative pictures of DAT scan in WT and *hASL* LNP‐mRNA treated *Asl* ^*Neo/Neo*^ mice. (C–E) Quantification of retention of ^123^I‐ioflupane in (C) whole brain, (D) uptake in locus coereleus and (E) in basal ganglia. (F) Schematic representing the synthesis and catabolism of serotonin and dopamine. (G–K) Metabolomic data of serotonin and dopamine catabolism pathways in WT and *hASL* LNP‐mRNA treated *Asl* ^*Neo/Neo*^ mice, (G) 5‐HIAA, (H) 3OMD, (I) dopamine, (J) DOPAC and (K) HVA. (L) Dopamine turnover estimated by (DOPAC + HVA)/dopamine. Graph shows mean ± SD. (C–E, G–L): Unpaired two‐tailed Student's *t* test; *n* = 5–6. AADC, aromatic L‐amino acid decarboxylase; BG, basal ganglia; COMT, catechol‐O‐methyltransferase; CT, computed tomography; DA, dopamine; DOPAC, 3,4‐dihydroxyphenylacetic acid; 5‐HIAA, 5‐hydroxyindolacetic acid; HVA, homovanillic acid; 3OMD, 3‐O‐methylDOPA; LC, locus coereleus; ns, not significant.

Individuals with ASA develop disabling motor symptoms with age. The pathophysiology involves the NO‐mediated downregulation of tyrosine hydroxylase (TH) and subsequent deficiency of central catecholamines.^21^ To date, different ASA mouse models have been described. The *Asl* ^*Neo/Neo*^ mouse is a hypomorphic mouse model, which presents with a systemic phenotype. The *Asl* ^*flox/flox*^ *;TH Cre* ^*+/−*^ mouse is a model of ASA, where the *Asl* gene is knocked out specifically in dopaminergic neurones. Both models exhibit motor abnormalities. Central catecholamine deficiency was demonstrated in *Asl* ^*flox/flox*^ *;TH Cre* ^*+/−*^ mice, but has not been assessed in *Asl* ^*Neo/Neo*^ mice. As *Asl* ^*Neo/Neo*^ mice recapitulate the human phenotype with systemic ASLD affecting cerebral cell types, we aimed to model and assess the dopaminergic pathway in vivo using this model. ^123^I‐ioflupane SPECT or dopamine transporter (DAT) scans of adult WT and adult *Asl* ^*Neo/Neo*^ mice which had been treated with *hASL* LNP‐mRNA from birth were compared. These, technologies are routinely used in clinical settings to study inherited^29^ or acquired neurodegenerative^30^ disorders affecting the dopamine synthesis or uptake pathway. Following IV administration of ^123^I‐ioflupane, no cerebral retention of the radiotracer was observed in either the WT and *Asl* ^*Neo/Neo*^ mice (Figure 4A,B). Analysis of ^123^I‐ioflupane retention index for the whole brain (Figure 4C), and uptake in locus coeruleus (Figure 4D) and basal ganglia (Figure 4E) did not show any difference.

To assess whether serotonin and dopamine catabolism pathways (Figure 4F) were abnormal in *Asl* ^*Neo/Neo*^ mice, cerebral dopamine‐related metabolites were measured in 2‐week‐old untreated *Asl* ^*Neo/Neo*^ mice and compared to those of WT mice. No significant difference was observed (Figure 4G–L). An increasing trend in cerebral 5‐HIAA levels was seen in *Asl* ^*Neo/Neo*^ mice compared to WT mice (*p* = 0.09) (Figure 4G).

## 4 DISCUSSION

This study sheds light on the natural history of neurodegenerative‐related symptoms, movement disorder, hypotonia/fatigue and abnormal behaviour, which are common but poorly characterised features in ASA chronic encephalopathy.^3^, ^21^ After a symptom‐free period during childhood, neurodegenerative‐related symptoms develop progressively during the second and third decades of life, affecting 29% of patients. Movement disorder including tremor and ataxia affected 15% of patients, which is comparable to 17% and 19% described previously by Baruteau et al. in a British cohort^3^ and Lerner et al. in an international consortium with patients from North‐America,^20^ respectively. These symptoms, especially intention tremor, are particularly disabling. Tremor is more characteristic of ASA when compared to other urea cycle defects.^20^ The main pathophysiological mechanism proposed is down‐regulation of TH, the initial and rate‐limiting step in the biosynthetic pathway of catecholamines including dopamine, noradrenaline and adrenaline. TH activity is altered by two mechanisms: decreased ASL expression mediated by NO‐mediated downregulation of TH transcriptional factor cyclic adenosine monophosphate response element‐binding protein, and abnormal protein conformation caused by deficient nitrosylation.^21^ The pathogenic role of central catecholamine deficiency in ASA has been shown in abnormal stress response, epilepsy, memory and movement disorder. These symptoms can be partially rescued by NO donors.^20^, ^21^

Neuroimaging in individuals with ASA at baseline has shown various non‐specific findings such as brain atrophy, focal infarcts, white matter and basal ganglia hyperintense signals, grey matter heterotopia, ulegyria and gliosis,^3^, ^12^ findings which can be associated with developmental delay and epilepsy. Focal infarct is occasionally a complication of hyperammonemia.^10^ In a significant number of cases (48%), neuroimaging with conventional MRI sequences is normal.^3^ Magnetic resonance (MR) spectroscopy can show reduced creatine or increased guanidinoacetate in white matter and reduced N‐acetylaspartate, suggesting reduced cellularity in basal ganglia.^3^ DTI enables a more accurate characterisation. In one patient with significant movement disorder reported here, brain MRI with conventional sequences appeared normal but DTI showed abnormal remodelling of grey and white matter affecting basal ganglia, especially the globi pallidi, and white matter tracts in the vicinity of these structures. Pallidi are essential structures in movement control. Basal ganglia, especially thalami, are sensitive to hyperammonemia^31^, ^32^ and abnormal signals in context of acute decompensations have been demonstrated previously.^33^, ^34^ Although only one case is presented here, this may be characteristic of this disorder as abnormal basal ganglia findings are rare during follow‐up of individuals with ASA with normal ammonia levels.^3^, ^12^ Our case report highlights that DTI is a better tool than conventional MRI when assessing neuroimaging abnormalities in individuals with ASA presenting with a movement disorder. It is possible to speculate that previous hyperammonemic decompensations in this patient are at least partially responsible for the DTI findings.^35^ Further work is needed to provide a definitive description of the nature and evolution of these abnormalities. Although this individual with ASA was involved in a clinical trial with liver‐directed cell therapy, no efficacy was observed, no improvement of ureagenesis was noted and the patient remained on the same combined therapy of diet and scavengers with similar dose. Interestingly, abnormal hyperintensities of basal ganglia with a rostro‐caudal gradient in the putamen have been described in individuals with ASA.^36^ Blood brain barrier dysfunction has been observed in ASLD mice.^36^ Whether some MRI findings observed in individuals with ASA could be attributable to enhanced blood brain barrier leakage warrants the need for additional studies.

We attempted to model the features observed in human neuroimaging in *Asl* ^*Neo/Neo*^ mice, which recapitulate the human disease phenotype and present with a motor and movement disorder. To decipher the cerebral consequences of central ASLD and avoid any confounding factors caused by hyperammonemia, we treated *Asl* ^*Neo/Neo*^ mice with liver‐targeting mRNA therapy to restore ureagenesis. *Asl* ^*Neo/Neo*^ mice show systemic NO deficiency,^37^ especially in the brain,^19^, ^38^ and are assumed to present with central catecholamine deficiency as observed in the knockout *Asl* model in dopaminergic neurons, *Asl* ^*flox/flox*^ *;TH Cre* ^*+/−*^ mouse.^20^, ^21^ Conventional and DTI sequences did not show any significant differences between LNP‐mRNA treated *Asl* ^*Neo/Neo*^ and WT mice. It could be that these abnormalities remain mild and are below the sensitivity of these neuroimaging tools. Additionally, liver‐targeting mRNA therapy could as well partially alleviate the severity of the neurological disease, as suggested in liver transplanted individuals with ASA.^15^ It is also possible that LNP‐mRNA treated *Asl* ^*Neo/Neo*^ mice scanned at 8 weeks of life might have developed abnormal neuroimaging findings later in life. The absence of differences observed between LNP‐mRNA treated *Asl* ^*Neo/Neo*^ and WT mice by cerebral ^123^I‐ioflupane SPECT suggests the persistence of dopaminergic neurons with no neurodegeneration and supports the findings of Lerner et al. with a functional reduction of central catecholamine synthesis, which responds to NO therapy.^20^, ^21^ Another scenario could be a compensatory mechanism, with an increased number of DAT receptors to palliate the reduction of dopaminergic neurons as observed in Parkinson's disease.^39^ Also, we cannot exclude the possibility that SPECT imaging performed later in life could show a different result. The absence of differences in metabolomic analysis of dopamine synthesis and catabolism pathways between LNP‐mRNA treated *Asl* ^*Neo/Neo*^ and WT mice suggests that TH downregulation is compensated for in this mouse model and does not affect the overall levels of central catecholamine metabolites. To better assess TH downregulation, measurement of dopamine metabolites and TH transcriptomics specifically in dopaminergic neurons rather than whole brain would provide a more accurate measurement. Overall, these findings bring hope for individuals with ASA with regard to liver replacement therapy, that is, liver transplantation or gene therapy, as they support a therapeutic window, where movement disorder could be responsive to adequate therapy like NO donors, with no or limited damage of dopaminergic neurons.

This work has limitations due to the small number of patients identified with this rare disease, the methodology with retrospective analysis, the findings of DTI MRI reported in only one patient and limited evidence of TH downregulation in *Asl* ^*Neo/Neo*^ mice. Our findings require further prospective of larger cohorts of patients with urea cycle defects, which could be achieved via existing registries in Europe and in the United States, and warrant the inclusion of DTI sequences in neuroimaging of individuals with ASA.^40^, ^41^ Additionally, to better decipher the complex neuropathophysiology of ASA, further characterisation of dopaminergic neurotransmission at the cellular or circuitry levels in *Asl* ^*Neo/Neo*^ mice would be required. Furthermore, developing surrogate models such as induced pluripotent stem cell derived neurons or three‐dimensional organoid cultures^42^, ^43^ or cultured ex vivo precision‐cut organ slices^44^, ^45^ could become valuable alternatives to screen therapies able to treat TH downregulation, suspected to be the main driver of this movement disorder in ASA.^21^

## 5 CONCLUSION

In conclusion, neurodegenerative‐related symptoms in ASA are a common and debilitating feature, which appear during adolescence and early adulthood. These symptoms are independent from the age of onset of hyperammonemia. DTI neuroimaging shows remodelling of basal ganglia, particularly globi pallidi, which provides an anatomical substratum for movement disorder. DTI neuroimaging, cerebral ^123^I‐ioflupane SPECT and cerebral dopamine metabolomics in *Asl* ^*Neo/Neo*^ mice with restored ureagenesis failed to identify endpoints which could be used for therapeutic testing to target movement disorder.

## AUTHOR CONTRIBUTIONS

Sonam Gurung and Julien Baruteau designed the project and wrote the manuscript. Stephanie Grunewald, Saketh Karamched, Dany Perocheau, Loukia Touramanidou, Claire Duff, Tammy L. Kalber conducted or assisted with animal experiment and neuroimaging. Kiran K. Seunarine, Christopher A. Clark, Jonathan D. Clayden analysed patient's neuroimaging. Tom Baldwin and Simon Eaton performed the gas chromatography mass spectrometry analysis of cerebral neurotransmitters. Philippa B. Mills and Paul Gissen provided assistance with ethical agreement. Nour Elkhateeb, Karolina M. Stepien, Reena Sharma, Andrew Morris, Thomas Hartley, Laura Crowther, Stephanie Grunewald, Maureen Cleary, Helen Mundy, Anupam Chakrapani, Spyros Batzios, James Davison, Emma Footitt, Karin Tuschl, Robin Lachmann, Elaine Murphy, Saikat Santra, Mari‐Liis Uudelepp, Mildrid Yeo, Julien Baruteau assisted with patients. Patrick F. Finn, Alex Cavedon, Summar Siddiqui, Lisa Rice, Paolo G. V. Martini, Andrea Frassetto provided LNP‐mRNA formulation for animal experimental work. Haya Alrashidi and Simon Heales measured central catecholamine levels. All authors reviewed and approved the final version of the manuscript.

## FUNDING INFORMATION

This study was supported by the United Kingdom Medical Research Council Clinician Scientist Fellowship MR/T008024/1 (to Julien Baruteau), NIHR Great Ormond Street Hospital Biomedical Research Centre (to Julien Baruteau) and research grant funding from Moderna Therapeutics. The views expressed are those of the author(s) and not necessarily those of the NHS, the NIHR or the Department of Health.

## CONFLICT OF INTEREST STATEMENT

Julien Baruteau is in receipt of research funding from Moderna Therapeutics. Patrick F. Finn, Alex Cavedon, Summar Siddiqui, Lisa Rice, Paolo G. V. Martini and Andrea Frassetto are employees of Moderna Therapeutics. Andrea Frassetto and Lisa Rice are inventors of patent application no. PCT/US23/17573 ‘Lipid nanoparticles and polynucleotides encoding argininosuccinate lyase for the treatment of argininosuccinic aciduria’. Other authors have no competing financial conflict of interest to declare.

## ETHICS STATEMENT

This research study was conducted retrospectively from medical notes. Participants' data were recorded anonymously. Informed consent approved by the National Research Ethics Service Committee London‐Bloomsbury (13/LO/0168) was obtained for participants and/or legal guardians for the following centres: Great Ormond Street Hospital for Children NHS Trust, National Hospital for Neurology and Neurosurgery, Evelina London Children's Hospital, Salford Royal NHS Foundation Trust, and Manchester Centre for Genomic Medicine. Birmingham Children's Hospital institutional review board advised that it was not necessary to consent participants at this site due to the collection of anonymous data.

## INFORMED CONSENT

Written consent for publication was obtained from the patient who underwent DTI neuroimaging.

## Supporting information


**Data S1.** Supporting information.

## Data Availability

The database is available in Table S1.
