# Supplementary material for: The incidence of movement disorder increases with age and contrasts with subtle and limited neuroimaging abnormalities in argininosuccinic aciduria
Source: J Inherit Metab Dis. 2023 Dec 4;47(6):1213–27. doi: 10.1002/jimd.12691 (PMC11586606; doi:10.1002/jimd.12691)
Supplement: Supplementary file 1 — Data S1. Supporting information. [file JIMD-47-1213-s001.docx]

**Supplementary table 1. Features of individuals with ASA included in this study**

F Female, M Male, N No, NA Not available, Y Yes.


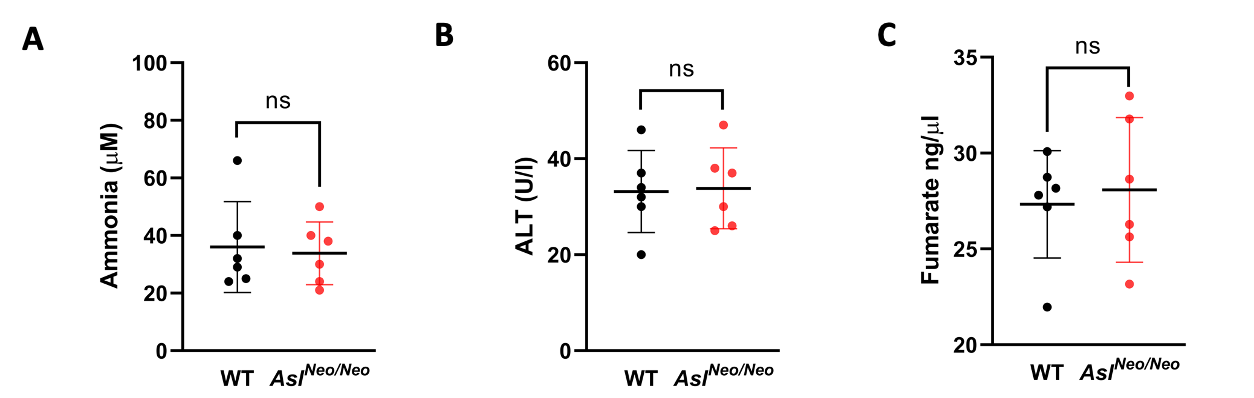


**Supplementary Figure 1. Weekly *hASL* mRNA therapy from birth corrects liver ureagenesis of *Asl^Neo/Neo^* mice.** (**A**) Average plasma ammonia and (**B**) plasma ALT levels from WT or *hASL* mRNA treated *Asl^Neo/Neo^* mice at harvest. (**C**) Liver ASL activity from WT and *hASL* mRNA treated *Asl^Neo/Neo^* mice at harvest. (**A-C**) Graph shows mean $\pm$SD. Unpaired 2-tailed Student’s t test; ns=not significant, n=6. ALT= Alanine aminotransferase.

**
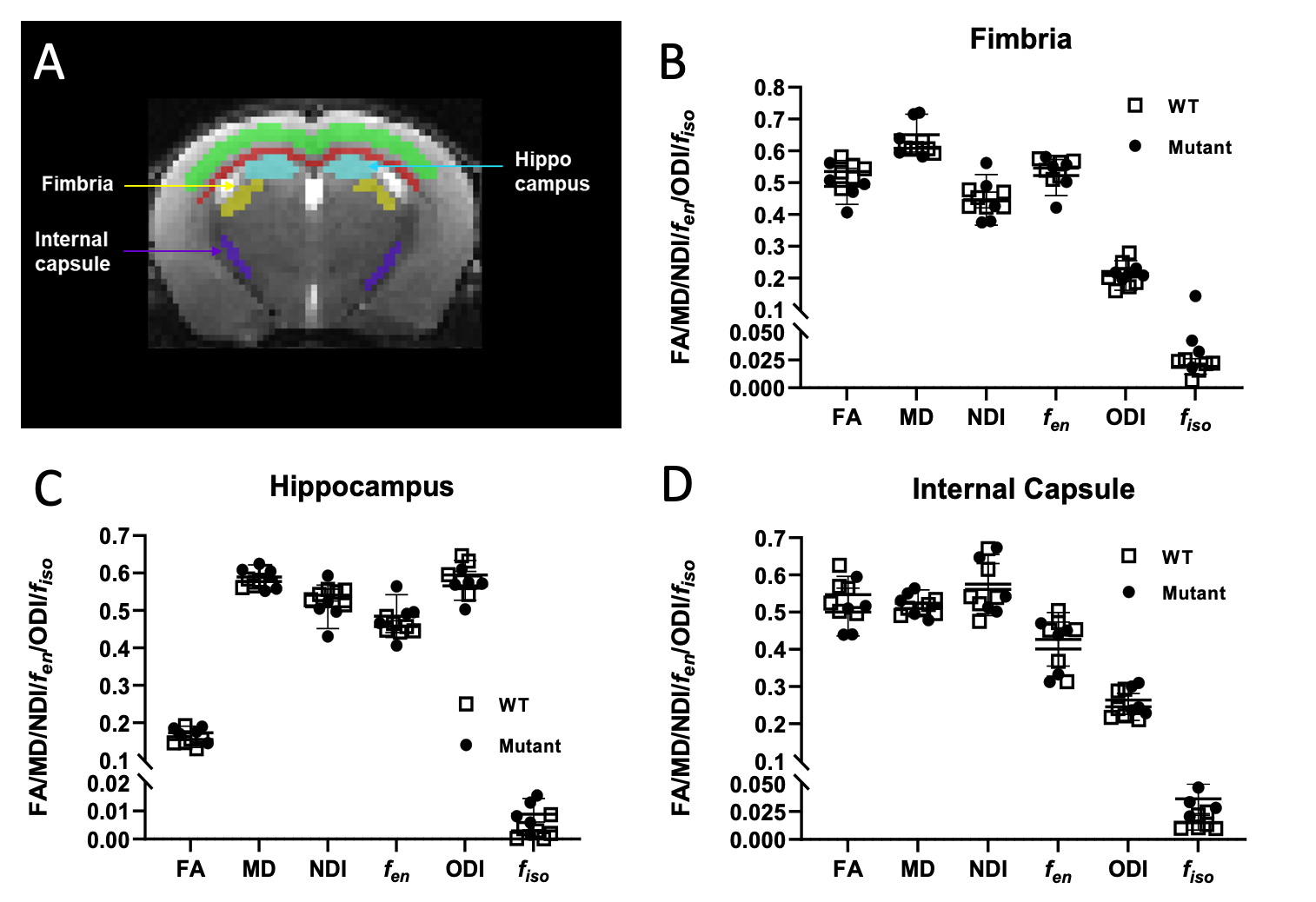
**

**Supplementary Figure 2. Functional MRI did not show involvement of regions of interest in LNP-mRNA treated ASA mice.** (**A**) Manual definition of cerebral regions of interest with ITK-SNAP software. (**B-D**) Analysis of fMRI endpoints in various cerebral regions of interest (**B**) fimbria, (**C**) internal capsule and (**D**) hippocampus. Graph shows mean $\pm$SD. (**G-I**): Unpaired 2-tailed Student’s *t* test (False Discovery Rate corrected; *p* = 0.05; ns=not significant, n=5-6.
